# Burnout in health-care professionals during reorganizations and downsizing. A cohort study in nurses

**DOI:** 10.1186/1472-6955-9-8

**Published:** 2010-06-04

**Authors:** Kirsten Nordang, Marie-Louise Hall-Lord, Per G Farup

**Affiliations:** 1 Dept. of Medicine, Innlandet Hospital Trust, Gjøvik, Norway; 2 Faculty of Health, Care and Nursing, Gjøvik University College, Gjøvik, Norway; 3 Dept. of Nursing, Karlstad University, Karlstad, Sweden; 4 Unit for Applied Clinical Research, Norwegian University of Science and Technology, Trondheim, Norway

## Abstract

**Background:**

Burnout is a psychological reaction triggered by interaction between personal characteristics and stress factors. Reorganizations and downsizing with increased workload imply stress for health-care professionals. This is a study of burnout in nurses during a period with two comprehensive reorganizations.

**Methods:**

In this quasi-experimental retrospective cohort study, burnout was assessed in nurses with long work experience in three surveys during a 30 months' period with two comprehensive reorganizations and downsizing of a hospital unit with mostly seriously ill patients with cancer. Burnout was measured with Bergen Burnout Indicator (BBI) at each survey, and "Sense of Coherence" (SOC) with Antonovsky's questionnaire at the last survey.

**Results:**

One man and 45 women aged 30 to 65 years were invited to the surveys. There was a significant increase in burnout during the study period, the mean increase in BBI-score was 12.5 pr year (p < 0.001). The proportion of satisfied nurses at the first and last survey were 84% and 35% respectively, and the proportions with burnout were 0% and 29% respectively (p < 0.001). Except for auxiliary nurses with experience from the medical department, all subgroups experienced a significant increase in BBI. Burnout was associated with low SOC (p < 0.001, r square 0.33).

**Conclusions:**

There was a significant development of burnout in a group of nurses during a period with two reorganizations and downsizing. Burnout was associated with low SOC. Working with seriously ill patients with cancer has probably made the nurses exceptionally vulnerable to the stress and workload related to the reorganizations.

## Background

Burnout syndrome is a well-known psychological reaction among health-care workers [1-3]. Triggering factors are personal characteristics such as low sense of coherence (SOC), close patient relations, workload, autonomy, professional development, performance feed-back, work-environment and interaction of these and other stress related strains [1,4-7]. Burnout usually takes some years to develop, but is less common in older employees with several years of experience [1,8,9].

Reorganizations and downsizing have become an increasingly common stressful event in the health-care system and have been associated with a high degree of depression, anxiety and emotional exhaustion [10,11]. A few studies have assessed burnout related to reorganization[12,13].

This retrospective cohort study aimed at studying the development of burnout in personnel with long work experience during two large reorganizations with downsizing, and factors like SOC, profession and work experience related to burnout. Concern for the nursing staff and interest for the human costs of repeated reorganizations motivated the study.

## Methods

### Participants

In one hospital unit with mostly cancer patients, all registered nurses and auxiliary nurses with permanent employment and more than five years' working experience were included. The group was selected because of low expected burnout potential.

### Design

The design is a quasi-experimental retrospective cohort study. The participants were followed for 30 months during two large reorganizations of the department. The purposes of both reorganizations were significant resource reduction without impaired health care. The workload increased. Three surveys were performed, one before the first reorganization, the second between the two reorganizations, and the third after the last one. The head of the department (author KN) initiated the surveys aiming at implementation of preventive initiatives if necessary.

The first survey was performed after three years of regular service of an oncological hospital unit consisting of an inpatient clinic with 17 beds and an outpatient clinic. At the first survey, there was no knowledge of the forthcoming reorganizations. The participants filled in questionnaires for evaluation of burnout, and demographics were obtained from the hospital's administrative computer system.

At the first reorganization, the specialized inpatient clinic for oncology was closed down and the outpatient clinic for oncology was reduced. The rest of the oncology unit was merged with a specialized surgical unit which also was significantly reduced, into a new common medical and surgical unit with a mixture of employees with medical (oncological) and surgical (ear-nose-throat and gynaecological) experience. In total 12 employees with less than 5 years' employment and therefore not included in this study, were relocated to other work or denied prolongation of temporary position.

The second survey, 21 months after the first one, was performed when normal service was established in the new department. There was no knowledge or suspicion of the second reorganization starting immediately afterwards.

**Figure 1:**
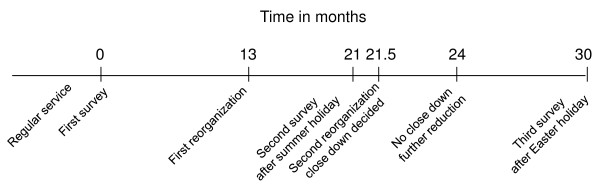
**The events related to time and holidays**.

The second reorganization started with a decision to close down the new department. After three months' operational planning with discharge and/or relocation of the employees, it was decided to continue with a reduced unit. None out of nine relocated or discharged employees with a short length of service participated in the study. At the third survey 9 months after the second one, when regular service was re-established anew, a questionnaire for measuring sense of coherence was added. Figure 1 shows the events related to time and holidays.

### Variables

Gender, age, profession, duration and type of work experience and size of current position were noted.

Burnout was measured with Bergen Burnout Indicator (BBI). The questionnaire has been developed and validated in Norway and has good psychometric properties (reliability and validity) and high correlation with more commonly used questionnaires like Maslach Burnout Inventory and Stress Burnout Scale for Health Professionals [14-17]. The original BBI had 25 questions with seven response alternatives from "completely agree" to "completely disagree" giving scores from 25 to 175 with high values indicating burnout. In a revised version, which was used in this study, the mid alternative is left out and the scoring is 1, 2, 3, 5, 6, and 7. The BBI score is categorized into seven groups from "Completely satisfied" to "Severe burnout", which are merged into three groups: "Satisfied", "Observant" and "Burnout" [14,17].

Antonovsky proposed the concept "Sense of Coherence" (SOC) [18]. SOC reflects a person's ability to cope with stressful situations in life. This is a rather stable personality trait in adulthood, and describes how comprehensible, manageable and meaningful life appears [18,19]. A high score indicates better coping with life-stress or situations and relates to better health outcomes. Antonovsky's 13-item questionnaire, which was used in this study, has been widely used and has good psychometric properties in Scandinavian translations [20]. Responses are measured on a seven-point scale and summed up to a total score with range 13 to 91.

### Statistics

The authorities required that data were made completely anonymous before being analyzed and published. We were not allowed to follow one person from one point of time to the next, and lack longitudinal data for each participant. Therefore, data were analyzed as if they were independent, and the analyses are conservative (biased) in the sense that the p-values are expected to be higher than they would have been from analyses based on methods for longitudinal data.

Chi-square (with linear-by-linear associations when appropriate) was used for the table analyses, Pearson's correlation analyses for the association between BBI and SOC, and linear regression analyses for the relations between BBI (dependent variable) and time (independent variable). P-values < 0.05 were considered statistically significant and the main results are given with 95% confidence intervals (CI).

### Ethics

The aims of the surveys were surveillance and protection of the employees against burnout, and not research. Participation was optional. After having been made anonymous, the head of the hospital and the Norwegian Data Inspectorate, represented by Privacy Ombudsman for Research at Oslo University Hospital, Norway, approved the data for research.

## Results

**Figure 2:**
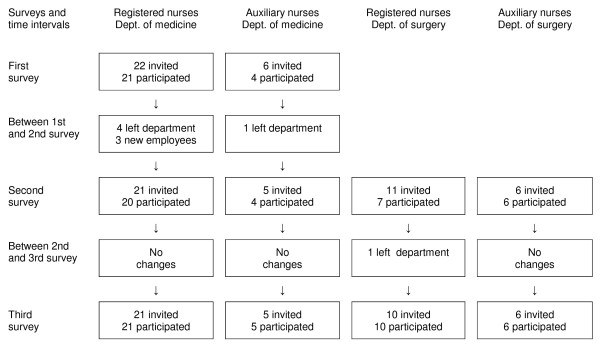
**The flow of participants during the study period according to profession and work experience**.

Forty-eight employees were invited to the surveys. Table 1 gives the characteristics of the subjects in the three surveys. Figure 2 shows the number of participants during the study divided into subgroups according to profession and work experience.

**Table 1:** Characteristics of the subjects in the three surveys.

Characteristics	First survey	Second survey	Third survey
Male/Female (no)	1/27	1/42	1/41
Age (years)	46 (30-65)	50 (33-63)	50.5 (34-64)
Duration of work experience (years)	17 (5-30)	21 (5-34)	22 (6-35)
Auxiliary Nurses/Registered Nurses (no)	6/22	11/22	11/31
Work experience: Medicine/Surgery (no)	28/0	26/17	26/16
Size of position (percentage)	75 (50-100)	75 (50-100)	75 (50-100)

The results are given as numbers (no) or median with range in brackets.

**Figure 3:**
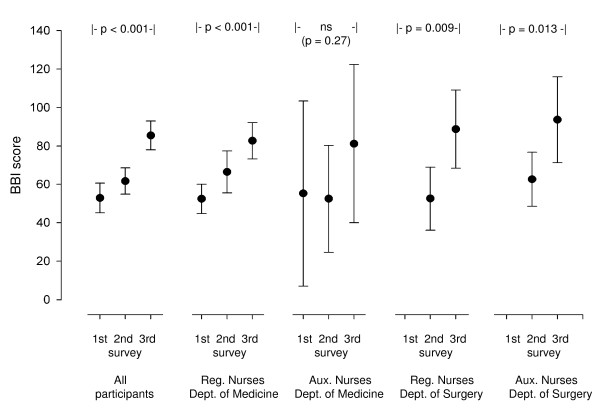
**The Bergen Burnout Indicator scores (BBI scores) (given as mean with 95% confidence interval) in the three surveys in all participants and in subgroups according to profession and work experience**. Aux. Nurses = Auxiliary nurses. Reg. Nurses = Registered nurses.

**Figure 4:**
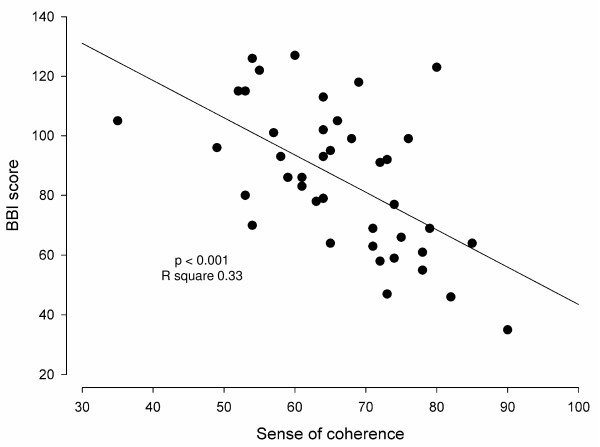
**The correlation between Sense of Coherence (SOC) measured with Antonovsky score and burnout measured with Bergen Burnout Indicator (BBI) at the third survey**. The correlation is statistically significant (p < 0.001, r square = 0.33).

Table 2 gives the BBI scores in the three surveys divided into predefined risk groups. There was a statistically significant deterioration in the BBI during the study (p < 0.001). Figure 3 shows the mean BBI scores with 95% confidence intervals in all participants and in subgroups according to profession and work experience. There was a statistically significant time-related deterioration of BBI score in the whole group of participants (p < 0.001, betta 12.5 BBI scores/year [CI 8.0; 17.0], adjusted R square 0.25) and in all subgroups except for the auxiliary nurses with work experience from medical departments. Mean SOC-score was 66.4 (SD 10.9). The correlation between high SOC and low BBI score at the third survey was statistically significant (p < 0.001; r square = 0.33) (figure 4).

**Table 2:** The Bergen Burnout Indicator (BBI) in the three surveys categorized into predefined risk groups.

	Satisfied				Burnout		
			
	Completely satisfied (25-29)	Very satisfied (30-49)	Fairly satisfied (50-74)	Observant (75-99)	Mild burnout (100-124)	Moderate burnout 125-149)	Severe burnout (150-175)
First survey	2 (8%)	9 (36%)	10 (40%)	4 (16%)	0 (0%)	0 (0%)	0 (0%)
Second survey	0 (0%)	12 (32%)	16 (43%)	5 (14%)	4 (11%)	0 (0%)	0 (0%)
Third survey	0 (0%)	3 (7%)	12 (29%)	15 (36%)	10 (24%)	2 (5%)	0 (0%)

The table gives the range of BBI score for each risk group. The results are given as number of subjects with proportion in brackets. The worsening of the BBI during the observation period (from the first to the last survey) was statistically significant (p < 0.001, exact linear-by-linear association).

## Discussion

The main finding was a marked and clinically significant increase in burnout score during the study period. The increase was statistically significant in the whole group of participants and in the subgroups with different professions and work experience. The only exception was a small group of auxiliary nurses with experience from medical departments. They showed the same order of deterioration, but due to a wide range in the results, the changes were not statistically significant.

For comparison, a Norwegian database with BBI scores from 3582 persons is available, nearly all were health-care professionals [17]. At the start of this survey, "Satisfaction" was reported by 84% of the participants compared with 55% in the Norwegian database, indicating a well-functioning group with long job experience and high degree of job satisfaction. At the end, the prevalence of "Satisfaction" had fallen to 36% and the prevalence of "Burnout" had increased from 0% to 29%, compared with 17% "Burnout" in the Norwegian database. Compared with health-care professionals in Norway, there was a change in the study group from a high degree of satisfaction to a high degree of burnout.

The time of registration probably affects the results. The first survey with a high degree of satisfaction was performed in a period with regular service and a well-functioning group without prior knowledge of forthcoming changes. The second and third surveys were deliberately performed after the summer and Easter holidays to reduce an acute, stress-related, blow off steam and an exaggerated response.

Burnout takes at least one to two years to develop, but normally longer [1,8]. The study period (30 months) is within the possible frame for developing burnout, but is rather short. The risk of burnout is highest at a young age and during the first working years; it seems as if personal maturation, protective psychological mechanisms and a selection of robust health-care professionals with realistic aims and preservation of work energy takes place with increasing age and work experience [8,9,21]. At entrance, the study group was robust with long work experience, a relatively high age and a high degree of satisfaction as judged by the BBI-score. The rapid development of burnout was therefore unexpected and judged as alarming.

Burnout is related to personal and external factors. Personal factors of importance are low self-esteem, unrealistic expectations, weak sense of coherence, excessive conscientiousness and high education level [1,2,4,22-24]. Uncertainty about continued employment, discrepancy between requirements and resources, large workloads, missing influence on own workplace, changes and uncertainties concerning procedures, structure and reporting are external factors related to burnout [1,5,7,10,11]. These factors affect job situation negatively during reorganizations and workforce reductions, as do reduced social and occupational support from colleagues and manager, teaching, training, coaching and counselling, which are also related to burnout [4,10,13,25,26].

In this study, the participants were working with seriously and incurably ill and dying patients with cancer. This type of work has been shown to be meaningful and personally stimulating for health-care professionals, but also stressful [2,3,5,27]. Cost reductions with no or minimal influence on the health care was the framework for the reorganizations. Downsizing results in discrepancy between requirements and resources, increased workload and impeded care and treatment, factors which are particularly difficult to accept and an extra stress factor for professionals working with terminally ill patients.

Sense of coherence (SOC) was measured at the last survey. In accordance with other studies, there was a significant association between burnout and low SOC [7,23,24]. Since SOC describes how comprehensible, manageable and meaningful life appears, persons with a strong SOC cope, as expected, better with stressful undesired events like reorganizations. Since SOC is a stable personality trait, it is likely that a low SOC at the first survey was a risk factor for burnout. The study does, however, not exclude that burnout caused low SOC since SOC might be sensitive to external influences over time [7].

The significant burnout seen during the 30 months' period was probably due to the comprehensive and rapid reorganizations that had an extraordinary high negative impact on nurses working with seriously and terminally ill patients with cancer. The finding that downsizing and reorganizations increased the risk of burnout clearly shows that implementation of initiatives to prevent burnout should be mandatory during such periods. The most efficient ways to prevent burnout under such conditions are largely unknown, and studies aimed at finding the best preventive methods are required.

### Strengths and weaknesses

The restricted number of participants might affect the external validity. There are no indications of a particularly vulnerable group of participants. On the contrary, it was a group with robust personality, long work experience and high degree of job satisfaction as measured with BBI before the reorganizations. However, working with seriously and terminally ill patients with cancer might have made them exceptionally vulnerable. The results are clear, convincing and statistically significant.

The comprehensive and frequent reorganizations in the hospital made it an ideal quasi-experimental situation for studies of effects of reorganizations and downsizing. The retrospective observational design has inherent weaknesses, but prospectively controlled trials are seldom possible.

A few participants quit their jobs during the study period and some new employees with work experience were engaged. This reduces the validity, but is inevitable in such observational studies and is likely to reduce rather than increase the degree of burnout. The high participation rate increases the validity. Separate analyses of subjects participating in all surveys would have been of interest but was impossible since data had to be made completely anonymous prior to the analyses and publication.

Measurement of SOC at all surveys would have given valuable information about changes in SOC during the stress period. Any influence from the head of the hospital unit, who performed the surveys, such as exaggeration or concealing of the problems, is unknown but unlikely.

## Conclusions

During a 30 months' period with two comprehensive reorganizations and downsizing, nurses with long experience and working with seriously and incurably ill and dying patients with cancer showed a significant and unexpectedly fast development of burnout. The reorganizations are the only likely explanation, but working with seriously and terminally ill patients with cancer might have hastened the development. Low SOC seemed to be a risk factor for burnout.

## Competing interests

KN was head of the department throughout all the reorganizations.

## Authors' contributions

KN has collected the data and has been the main contributor to the concept and design of the study, has taken part in the analyses of the data, made the first draft manuscript, has thoroughly revised the manuscript and is the main responsible for the professional content. MH has taken part in the design of the study, participated actively in the analyses and interpretations of the data, has supervised the writing and revised the manuscript. PF has taken part in the design of the study, is responsible for the statistical analyses and interpretation of the results, and has drafted and written the final manuscript. All authors approved the published version.

## Pre-publication history

The pre-publication history for this paper can be accessed here:

http://www.biomedcentral.com/1472-6955/9/8/prepub
